# Perceived need to increase physical activity levels among adults at high risk of type 2 diabetes. A cross-sectional analysis within a community-based diabetes prevention project FIN-D2D

**DOI:** 10.1186/1471-2458-12-514

**Published:** 2012-07-10

**Authors:** Kati Vähäsarja, Sanna Salmela, Jari Villberg, Pauli Rintala, Mauno Vanhala, Timo Saaristo, Markku Peltonen, Sirkka Keinänen-Kiukaanniemi, Eeva Korpi-Hyövälti, Urho M Kujala, Leena Moilanen, Leo Niskanen, Heikki Oksa, Marita Poskiparta

**Affiliations:** 1 Department of Health Sciences, University of Jyväskylä, Jyväskylä, 40014, Finland; 2 Department of Sport Sciences, University of Jyväskylä, Jyväskylä, 40014, Finland; 3 Family Practice Unit, Central Hospital Central Finland, Jyväskylä, 40620, Finland; 4 Family Practice Unit, Kuopio University Hospital, Kuopio, 70210, Finland; 5 University of Eastern Finland, Kuopio, 70211, Finland; 6 Finnish Diabetes Association, Tampere, 33680, Finland; 7 Pirkanmaa Hospital District, Tampere, 33680, Finland; 8 National Institute for Health and Welfare, Helsinki, 00271, Finland; 9 Institute of Health Sciences, University of Oulu, Oulu, 90014, Finland; 10 Unit of General Practice, Oulu University Hospital, Oulu, 90220, Finland; 11 Health Centre of Oulu, Oulu, 90100, Finland; 12 Department of Internal Medicine, Seinäjoki Central Hospital, Seinäjoki, 60220, Finland; 13 Department of Medicine, Kuopio University Hospital, Kuopio, 70210, Finland; 14 Department of Medicine, Central Hospital Central Finland, Jyväskylä, 40014, Finland; 15 University of Eastern Finland, Kuopio, 70211, Finland; 16 Tampere University Hospital, Tampere, 33521, Finland

**Keywords:** Physical activity, Exercise, Behaviour change, Perception, Awareness, Type 2 diabetes, Prevention, FIN-D2D, Lifestyle intervention

## Abstract

**Background:**

Increased physical activity is a cornerstone of type 2 diabetes prevention. The perception of a need to change is considered essential in behaviour change processes. However, the existing literature on individuals’ perceived need to change health behaviour is limited. In order to improve understanding of diabetes prevention through increased physical activity levels (PAL), we assessed factors associated with perceiving a need to increase PAL among adults at high risk of diabetes.

**Methods:**

Opportunistic screening was used within a primary-care based lifestyle intervention covering 10 149 men and women at high risk of type 2 diabetes. Data were obtained at baseline visits. The explored determinants were demographic, anthropometric/clinical, behavioural and psychosocial characteristics, along with four categories of PAL awareness. Logistic regression was used in the analysis.

**Results:**

74% of men (n = 2 577) and 76% of women (n = 4 551) perceived a need to increase their PAL. The participants most likely to perceive this need were inactive, had a larger waist circumference, rated their PAL as insufficient, and were at the contemplation stage of change. Smoking, elevated blood pressure, dyslipidaemia, and a family history of diabetes were not associated with this perception. The likelihood was also greater among women with less perceived fitness and less education. Demographic factors other than education did not determine participants’ perceived need to increase PAL. PAL overestimators were less likely to perceive the need to increase their PAL than realistic inactive participants.

**Conclusions:**

Subjective rather than objective health factors appear to determine the perception of a need to increase PAL among adults at high risk of diabetes. Client perceptions need to be evaluated in health counselling in order to facilitate a change in PAL. Practical descriptions of the associations between metabolic risk factors, PAL, and diabetes are needed in order to make the risk factors concrete for at-risk individuals.

## Background

Extensive literature has indicated that type 2 diabetes can be prevented or delayed by increased levels of physical activity (PAL) [1-8]. People with diabetes are claimed to be least ready to increase their physical activity levels (PAL), to a greater degree than people without diabetes or people with heart disease, elevated blood pressure, or dyslipidaemia [9]. However, inclinations toward PAL behaviour change among the high-risk population remain imperfectly understood. It has been suggested [10,11] that perceived risk will increase protective behaviour. Indeed, the vast majority of participants at risk of diabetes have been reported as intending to increase their PAL [12], or as having tried to increase their PAL during the past twelve months [13]. However, it has been reported elsewhere that diabetes risk perception is not associated with physical activity intention among adults at risk of type 2 diabetes [14], and that persons with a higher diabetes risk perception do not have a greater intention than others to increase their PAL [15].

An individual’s perceived need to change behaviour is regarded as a pre-step toward an intention [16-18]. However, only a few studies have assessed the perceived need to change health behaviour: three studies found perceived need to significantly predict the intention to improve diet [19-21], whereas no association was found between the perceived need and an intention to exercise [22]. The authors examined the perceived need to change health behaviour as a continuum within the *theory of planned behaviour*[23], taking the view that perceived need reflects perceived benefits, but that the perceptions of need and benefits are distinct. It was assumed that a person may have a positive attitude toward a behaviour, but not necessarily feel a need to change the behaviour if (i) the costs outweigh the benefits, (ii) the outcome of the behaviour is not valued highly by the person, or (iii) if the outcome is believed to be achievable by other means.

The concept of perceived need has been also associated with the *transtheoretical theory* (TTM) [17,24]. TTM suggests that individuals go through five distinct stages according to their motivational readiness to change behaviour. Individuals at the *precontemplation* stage are not regularly physically active, do not view their behaviour as a problem, and may therefore not perceive the need to change their behaviour. Once an individual becomes aware of his/her problem behaviour he/she may shift to the *contemplation* stage and continue to the *preparationaction,* and *maintenance* stages.

The third approach emphasising the role of perceived need in the change process is a counselling approach called *motivational interviewing* (MI) [18,25]. MI focuses primarily on enhancing the intrinsic motivation to change risk behaviour among individuals at the precontemplation and contemplation stages (who are reluctant or hesitative about the change). MI proposes that motivation to change increases in parallel with the individual’s own arguments regarding his/her perceived *needdesire, ability,* and *reasons* for change. These arguments strongly predict commitment, which in turn predicts change [26-28].

TTM [29-32] and MI [33,34] have both been widely applied to physical activity, and to type 2 diabetes prevention [35,36]. Even though these approaches regard a person’s perceived need to change as an essential preparatory step for the intention and commitment to behaviour change, the concept has rarely been examined. Similarly, the existing literature on the correlates of the pre-contemplation stage is limited, and has tended to focus mainly on psychosocial aspects [15,37,38].

Perceiving the need to increase PAL is crucial for a population at high risk of type 2 diabetes. Thus, it is important to explore which factors determine this need. We examined demographic, anthropometric/clinical, behavioural, and psychosocial factors as determinants of this need perception among adults at high risk of type 2 diabetes. Since previous studies have shown misperception of one’s own physical activity level to be a common phenomenon, and also to be associated with physical activity intention [37,39-43], we further examined the perceived need to increase PAL across four categories of physical activity awareness groups.

## Methods

### Study design and participants

FIN-D2D, an implementation project within a national programme for the prevention of type 2 diabetes, was conducted by the Finnish Diabetes Association in five Finnish hospital districts covering a population of 1.5 million [44,45]. The collaborating bodies were the National Public Health Institute, the Ministry of Social Affairs and Health and the FIN-D2D Study Group. The specific aims were to improve the screening of people at risk of diabetes and to detect undiagnosed diabetes. The programme included intensified interventions among high-risk individuals as part of normal clinical practice (for the full study protocol see [46,47]).

Opportunistic screening was conducted in health care centres and pharmacies, and at various public venues and events (e.g. health fairs), in order to screen individuals at high risk of type 2 diabetes. A modified Finnish diabetes risk score (FINDRISC) was used for the screening process [48]. Individuals were referred to primary care for lifestyle intervention on a voluntary basis if they met any of the following criteria (i) a FINDRISC test score ≥15, (ii) a history of gestational diabetes, (iii) a history of impaired glucose tolerance or impaired fasting glucose, or (iv) a history of coronary heart disease. After identification, consenting high-risk individuals attended health check-ups conducted in primary health care units as part of standard care. Individuals received written information on the FIN-D2D and their participation in the project within normal primary care activities.

The present study analysed the baseline data from the FIN-D2D intervention, collected in 400 primary health care centres and occupational health clinics between 2004 and 2008. Altogether, 10 149 individuals aged 18–87 participated in the baseline assessments. Out of these, 9 984 individuals finally met the criteria for being at high risk. Glucose tolerance [49] was assessed with an oral glucose tolerance test (OGTT) among 8 353 of the participants. Individuals with previously-diagnosed type 2 diabetes, and screen-detected type 2 diabetes were excluded from the analysis, leaving 7 128 individuals in the analysis. The participant characteristics are presented in Table 1.

**Table 1:** Characteristics of the study population

	**Men (n = 2 577)**	**Women (n = 4 551)**
	**%**	**%**
Age (years)		
<45	14	13
45-54	32	30
55-64	20	21
≥65	34	35
Marital status***		
Married/cohabiting	77	72
Other	23	28
Educational level		
Low	41	39
Intermediate	50	53
High	9	9
Occupational status***		
Manual work	34	9
Non-manual work	25	44
Retired	34	35
Not employed	7	13
Family history of diabetes^1^***	57	66
Elevated blood pressure^2^***	88	82
Dyslipidaemia^3^***	30	40
Obesity^4^***	57	63
Waist circumference^5^***		
Normal	8	3
Elevated risk	22	10
High risk	70	87
Currently smoking^6^***	23	15
Physically inactive^7^	65	65
Self-rated fitness*		
Low	22	25
Satisfactory	50	48
High	28	27
Stage of change***		
Precontemplation	11	7
Contemplation	29	24
Preparation	39	46
Action	12	14
Maintenance	9	9
Physical activity level perceived as sufficient*	33	30
Perceives the need to increase physical activity level	74	76

^1^ At least one of the first-degree relatives had diabetes (type 1 or 2).

^2^ Systolic blood pressure ≥130 mmHg and/or diastolic blood pressure ≥85 mmHg.

^3^ HDL < 1.03 mmol/l (men), <1.29 mmol/l (women); LDL ≥ 3 mmol/l or/and triglycerides ≥ 1.7 mmol/l, or medication for these lipid abnormalities.

^4^ Body mass index (BMI) ≥30 kg/m^2^.

^5^ Normal: <94 cm (m), <80 cm (w); elevated risk: 94-101 cm (m), 80-87 cm (w); high risk: ≥102 cm (m), ≥88 cm (w).

^6^ Includes occasional and regular smokers.

^7^ Engaging in physical exercise <30 minutes three times a week.

* *p* < 0.05 (for the statistical difference between the sexes).

** *p* < 0.01.

*** *p* <0.001.

### Measurements

The participants completed a questionnaire (questionnaire 1, issues regarding information on e.g. health behaviour and health status) and underwent health examinations. After these assessments the participants discussed with a nurse their health examination results, current health status, and health behaviour, in relation to current health recommendations and the risk of diabetes.

#### Perceived need to increase PAL

During the nurse-participant discussion, the nurse asked participants whether they perceived a need to change their health behaviours (to increase PAL, make changes in diet, quit smoking, reduce alcohol consumption, or lose weight in general). The nurses recorded the participants’ behaviour change targets within the structured questionnaire (response options “no need perceived”/ “need perceived”, for each item separately) according to the participant’s perception (questionnaire 2). The evaluation form (based on TTM and MI) was developed especially for the FIN-D2D intervention [46].

#### Demographic factors

The participants were classified into four age-group categories (see Table 1). The highest level of education was asked, with subsequent regrouping into three educational levels. The occupational status category originally included seven response-options, but these were subsequently regrouped into four categories. Marital status was also assessed.

#### Anthropometric and clinical risk factors

A family history of diabetes was assessed via a self-report. If at least one of the first-degree relatives (father, mother, or sibling) had diabetes (type 1 or type 2), family history was considered to be positive. Height and weight (usual light clothing, no shoes) were measured by the nurse for calculation of BMI (kg/m²). The participants were classified into two groups by BMI (<30 kg/m^2^ and ≥30 kg/m^2^). Waist circumference was measured to the nearest centimetre and classified into three groups: normal (men <94 cm, women <80 cm), elevated risk (men 94-101 cm, women 80-87 cm), and high risk (men ≥102 cm, women ≥88 cm). Blood pressure (mmHg, to the nearest 1mmhg) was measured twice from the right arm in sitting position (with at least a 1-min interval). The mean reading was recorded. Blood pressure was classified into two groups: normal, and elevated (systolic ≥130 mmHg and/or diastolic ≥85 mmHg) blood pressure. Plasma lipids and lipoproteins were determined locally from fasting venous plasma samples using enzymatic methods. Participants with impaired values of HDL (<1.03 mmol/l in men, <1.29 mmol/l in women), LDL (≥3 mmol/l)), or triglycerides (≥1.7 mmol/l), or medication for these lipid abnormalities were combined into a group called “dyslipidaemia”. The cut-off points used in the analysis were all based on international definitions [50,51].

#### Behavioural factors

Self-reported PAL was assessed with following questions (i) “How many times a week do you engage in leisure-time physical exercise causing you at least moderate sweating or breathlessness?” (ii) “For how long do you usually engage in leisure-time physical exercise at a time?”. The respondents were also asked to describe with structured descriptions their leisure-time physical activity during a typical week. Here the response options ranged from inactivity through light physical activity, and further to moderate/vigorous physical activity. The participants were classified as active if they did at least 30 minutes of physical exercise three times a week, and if they described the intensity of their usual leisure physical activity as at least moderate. All other participants were classified as inactive. Current smoking (including regular and occasional smoking) was assessed and dichotomised (*yes/no*).

#### Psychosocial factors

Participants rated their physical fitness via five response options ranging from *very high* to *very low*. Extremes were combined with the nearest options. Participants also rated their readiness to increase their PAL level via a 5-item question. Here the response options covered stages of change from the precontemplation to the maintenance stage. Furthermore, participants rated their current PAL as sufficient or as insufficient for maintaining their health and physical fitness (*yes/no*).

#### Physical activity awareness

PAL awareness was assessed by comparing the participants’ self-perceived sufficiency of their PAL with their self-reported PAL. Participants were classified into four physical activity awareness categories; *realistic active* (active by self-reported PAL, and perceiving their own PAL as sufficient), *overestimators* (inactive by self-report, but perceiving their own PAL as sufficient), *realistic inactive* (inactive by self-report, and perceiving their own PAL as insufficient) and *underestimators* (active by self-report, but perceiving their own PAL as insufficient).

### Statistical methods

SPSS for Windows (14.1) was used for the statistical analysis. Descriptive statistics were used to describe the sample. Pearson’s Chi Square tests were used to analyse differences between the groups and for a preliminary assessment of the associated factors. A multivariable logistic regression model was used to evaluate the association between a perceived need to increase PAL and demographic factors, anthropometric and clinical risk factors, behavioural factors, and psychosocial factors (Table 2). The results are presented as adjusted odds ratios (OR) and 95% confidence intervals (CI). Medication for a risk condition was taken as equivalent to having a risk condition. The category of lowest risk with regard to type 2 diabetes was used as a reference group for all variables. A separate logistic regression was used to determine the associations of physical activity awareness groups with the perceived need to increase PAL (Table 3).

**Table 2:** Multivariable logistic regression model for perceived need to increase physical activity levels (PAL) among adults at high risk of diabetes (n = 7 128) by selected variables

	**Men**			**Women**		
	**OR**	**(95% CI)**	** *p* **	**OR**	**(95% CI)**	** *p* **
Age (years)						
<45	1.00			1.00		
45-54	1.05	(0.65-1.66)	0.892	0.79	(0.55-1.15)	0.213
55-64	0.68	(0.41-1.12)	0.132	0.76	(0.51-1.14)	0.187
≥65	0.90	(0.51-1.58)	0.714	0.69	(0.44-1.08)	0.101
Marital status						
Married/cohabiting	1.00			1.00		
Other	0.95	(0.68-1.32)	0.771	0.95	(0.75-1.20)	0.665
Educational level						
High	1.00			1.00		
Intermediate	0.63	(0.35-1.13)	0.118	1.46	(1.01-2.09)	**0.042**
Low	0.60	(0.33-1.09)	0.092	1.49	(1.01-2.19)	**0.043**
Occupational status						
Non-manual work	1.00			1.00		
Manual work	0.91	(0.62-1.33)	0.628	0.78	(0.54-1.15)	0.209
Retired	0.70	(0.45-1.10)	0.119	0.84	(0.62-1.16)	0.295
Not employed	0.75	(0.42-1.35)	0.343	0.90	(0.64-1.27)	0.543
Family history of diabetes^1^						
No	1.00			1.00		
Yes	0.98	(0.75-1.28)	0.865	1.09	(0.88-1.36)	0.422
Elevated blood pressure^2^						
No	1.00			1.00		
Yes	0.95	(0.62-1.46)	0.821	0.84	(0.64-1.10)	0.208
Dyslipidaemia^3^						
No	1.00			1.00		
Yes	1.14	(0.85-1.55)	0.384	1.11	(0.90-1.38)	0.324
Body mass index (kg/m^2)^						
<30	1.00			1.00		
≥30	1.31	(0.94-1.83)	0.117	1.21	(0.95-1.55)	0.117
Waist circumference^4^						
Normal	1.00			1.00		
Elevated	1.34	(0.80-2.25)	0.273	1.37	(0.73-2.56)	0.322
High risk	1.83	(1.07-3.13)	**0.028**	2.72	(1.50-4.93)	**<0.001**
Smoking						
No	1.00			1.00		
Yes^5^	1.05	(0.75-1.47)	0.783	0.77	(0.58-1.04)	0.084
Physically active^6^						
Yes	1.00			1.00		
No	1.46	(1.07-1.98)	**0.017**	1.57	(1.57-1.98)	**<0.001**
Self-rated fitness						
High	1.00			1.00		
Satisfactory	1.04	(0.75-1.42)	0.833	1.30	(1.02-1.66)	**0.034**
Low	1.42	(0.90-2.22)	0.128	1.65	(1.18-2.33)	**0.004**
Stage of change						
Maintenance	1.00			1.00		
Action	2.17	(1.25-3.78)	**0.006**	2.29	(1.54-2.41)	**<0.001**
Preparation	2.54	(1.51-4.28)	**<0.001**	2.24	(1.56-3.23)	**<0.001**
Contemplation	2.68	(1.55-4.65)	**<0.001**	2.91	(1.90-4.46)	**<0.001**
Precontemplation	0.95	(0.53-1.69)	0.856	1.42	(0.87-2.31)	0.163
PAL perceived as sufficient						
Yes	1.00			1.00		
No	2.40	(1.72-3.37)	**<0.001**	2.95	(2.27-3.83)	**<0.001**

Adjusted odds ratios (OR), and their 95% confidence intervals (CI), and *p*-values

^1^ At least one of the first-degree relatives had diabetes (type 1 or 2).

^2^ Systolic blood pressure ≥130 mmHg and/or diastolic blood pressure ≥85 mmHg.

^3^ HDL < 1.03 mmol/l (men), <1.29 mmol/l (women); LDL ≥ 3 mmol/l or/and triglycerides ≥ 1.7 mmol/l, or medication for these lipid abnormalities.

^4^ Normal: <94 cm (m), <80 cm (w); elevated risk: 94-101 cm (m), 80-87 cm (w); high risk: ≥102 cm (m), ≥88 cm (w).

^5^ Includes occasional and regular smokers.

^6^ Engaging in physical exercise <30 minutes three times a week.

**Table 3:** **Perceived need to increase physical activity levels (PAL) by physical activity awareness categories, odds ratios (OR), 95% confidence intervals (CI), and *p* -values by sex**

	**Men**			**Women**		
	**OR**	**95% CI**	** *p* **	**OR**	**95% CI**	** *p* **
Realistic active^1^	1.00			1.00		
Overestimators^2^	1.51	(1.06-2.17)	**0.024**	1.79	(1.35-2.37)	**<0.001**
Realistic inactive^3^	7.14	(5.38-9.48)	**<0.001**	7.86	(6.37-9.70)	**<0.001**
Underestimators^4^	4.23	(2.92-6.14)	**<0.001**	4.68	(3.57-6.13)	**<0.001**

^1^ Defined as physically active (≥30 min 3 times a week) and perceiving PAL as sufficient.

^2^ Defined as physically inactive (<30 min 3 times a week), but perceiving PAL as sufficient.

^3^ Defined as inactive and perceiving PAL as insufficient.

^4^ Defined as active, but perceiving PAL as insufficient.

### Ethical considerations

The Ministry of Social Affairs and Health in Finland gave permission to the National Institute for Health and Welfare (formerly National Public Health Institute) to collect the data from health care units for evaluation purposes. In addition, the Institutional Review Board (IRB) of the National Institute for Health and Welfare approved the study. As lifestyle interventions were conducted as part of normal routine in primary health care units, informed consent was not collected from the participants; however, participants did receive written information on the FIN-D2D. The FIN-D2D project was not a scientific study under the legislation of the Medical Research Act in Finland, but rather an *implementation study within routine primary care in Finland.* Therefore, it would not in fact have been possible to collect statements of consent. Furthermore, the FIN-D2D data collection system was established by the participating hospital districts as part of normal patient records within primary health care.

## Results

The mean age of the participants was 55.4 (SD ± 10.23) years, and the mean BMI 31.3 kg/m^2^ (SD ± 4.7 kg/m^2^) in men, and 32.1 (SD + −5.4 kg/m^2)^ in women. The majority (64%) of the 7 128 participants were women. The basic characteristics of the participants are presented in Table 1. Smoking and elevated blood pressure were significantly more prevalent among men, whereas a family history of diabetes, dyslipidaemia, and obesity were all more prevalent among women. In total, 65% of the participants were classified as physically inactive. Women rated their fitness as low more often than men. However, compared to women, men were significantly more often (40% for men vs. 31% for women, *p* < 0.001) in the early stages of change (precontemplation and contemplation), and men also perceived their PAL as sufficient significantly more often than women (33% for men vs. 30% for women, *p* < 0.05).

In total, 74% of the men and 76% of the women perceived a need to increase their PAL with no significant difference between the sexes (Table 1). As shown in Table 2, a lower education level increased the likelihood of perceiving the need to increase PAL among women (intermediate education level: OR 1.46 [95% CI 1.01-2.09]; low education level: OR 1.49 [95% CI 1.01-2.19]). The opposite association appeared to exist among men, but did not reach statistical significance. Other demographic factors did not make any contribution. The need to increase PAL was significantly more often perceived by men and women with high-risk waist circumference than by those with normal waist circumference. The other anthropometric or clinical risk factors did not emerge as significant determinants. Physically inactive men and women were more likely than physically active participants to perceive the need to increase their PAL. In women, lower self-rated fitness also increased the likelihood of perceiving the need to increase PAL. Furthermore, men and women at the contemplation, preparation and action stages – but not at the precontemplation stage – were significantly more likely to perceive the need to increase their PAL than those at the maintenance stage of change; the perceived need to increase PAL was most likely among the participants at the contemplation stage and least likely among the participants at the precontemplation stage. The need to increase PAL was perceived more often by participants (both men and women) who rated their PAL as insufficient than by those who perceived their PAL as sufficient.

Out of the total population, 21% of men and 20% of women were classified as realistic active, 52% men and 55% women as realistic inactive, 13% men and 10% women as overestimators, and 14% men and 15% women as underestimators (not presented in the tables). In comparison with the realistic active participants, with regard to the three other awareness groups, the realistic inactive participants were the most likely to perceive the need to increase their PAL (Table 3). Overestimators had a significantly lower likelihood of perceiving a need to increase their PAL than realistic inactive participants.

## Discussion

The vast majority of the study participants (74% of men and 76% of women) perceived a need to increase their PAL. This reflects the high value placed on physical activity among the type 2 diabetes at-risk population, as indicated also by previous studies [12,13]. Considering the importance of increased physical activity in diabetes prevention, and the high prevalence of inactivity among the study population, the finding is promising, bearing in mind also that expression of a perceived need to change is a key component of a client’s “change talk”, predicting a commitment to change health behaviour [27,28]. Through professional advice [52] and effective counselling techniques e.g. [53] those perceiving the need to increase PAL could be encouraged to make a genuine commitment to changing their behaviour.

The present study showed that people at the contemplation stage of change, who perceived their PAL as sufficient, and who were classified as physically inactive, were more likely than others to perceive a need to increase their PAL. In addition, women who gave lower ratings to their personal physical fitness were more likely than others to perceive this need. A previous study [22] found general health and well-being, physical fitness, and weight control to be the most important determinants of a perceived need to exercise. Along similar lines, the present paper indicated a significant association between increased waist circumference and a perceived need to increase PAL in both sexes. By contrast, other type 2 diabetes risk factors did not contribute to this perception. These findings indicate the groups which are least likely to increase their PAL, and which should therefore be given particular attention within health care, in terms of endeavours towards diabetes prevention. Increased PAL would be of great benefit to individuals with a family history of diabetes, elevated blood pressure, or dyslipidaemia [8], yet the benefit does not appear to be recognised by these people. Waist circumference is an objective measure which is highly tangible for the individual, and which inevitably reflects the person’s current PAL in a manner readily apparent to that individual.

Our results suggest that among the high-risk population the need to increase PAL is strongly determined by the individual’s subjective perceptions. This finding is in line with the principles of the motivational interviewing approach to counselling, which emphasise the importance of listening to and reflecting individuals’ perceptions if one is to evoke the motivation to change. However, clients’ perceptions may easily be overlooked in a busy primary health care practice, especially if health professionals view the client as reluctant to change [54]. We would argue that if the perceptions of clients are neglected in health counselling, an important opportunity to support the client’s motivation for behaviour change will remain unutilised.

Our results support previous findings on PAL awareness indicating that overestimation of one’s PAL may be an obstacle to behaviour change [37,39-43]. In demonstrating a lack of any intention to change, the overestimators differed only slightly from realistic active persons, but there was a large difference between the overestimators and the realistic inactive persons. If the overestimators could be led to view their PAL realistically, they could be expected to see more clearly the need for change. Such efforts could also help to prevent incorrect tailoring in physical activity counselling [55].

As far as we know, no other studies have so far simultaneously assessed such a variety of determinants of the perceived need to increase PAL. One could expect that factors associated with a failure to perceive a need for increased PAL would be in line with determinants of the pre-contemplation stage of change. However, we found no evidence to support this presumption [37,38,56]. It appears that the pre-contemplation stage can best be regarded as a complex entity with its own set of determinants. It seems to be the case that some individuals at the precontemplation stage simply do not perceive the need to change their behaviours [57]. However, others perceive the need to increase their PAL in principle, yet – for whatever reason – do not consider changing their behaviour.

A previous study by Payne et al. [22] conducted among the general population (n = 286) found that 94% of the participants perceived the need to exercise (note that the authors did not assess the need to *increase exercise*, merely the need to *exercise* in general). It is true that the authors did not find a significant association between the perceived need and the intention or behaviour; yet the concept of perceived need requires further examination. Payne et al. assessed short-range intention only (the intention to exercise next week, and actual exercise behaviour a week later). Here it is worth bearing in mind that e.g. in the Diabetes Prevention Programme (DPP) [12], those who were at the contemplation stage at the baseline actually continued to increase their PAL until the 3-year (final) follow-up assessments; by contrast, other groups decreased their PAL. In fact, even six months can be regarded as a short period for changing physical activity behaviour [58-60]. The causal relationships between the perceived need to change, intentions, and behaviour should be assessed in future studies.

The study has some limitations. The study population is considered to represent the Finnish high-risk population [61]. Nevertheless, as opportunistic screening was used in the programme, there is a possibility of selection bias. The study participants were aware of their risk due to health examinations, and this aspect limits comparisons with the unaware high-risk population outside the programme. Furthermore, the assessment of the perceived need to change health behaviours was based on an evaluation form developed for the FIN-D2D intervention. The nurses were instructed to record not their own, but merely the participant’s perception; however, it is possible that a persuasive style on the part of the nurse could have influenced the responses (though attempts were made to avoid such a bias through careful instruction of the nurses). Furthermore, the self-reporting method of measuring physical activity limits the assessment of PAL and PAL awareness, due to issues of recall and response bias (e.g. social desirability, inaccurate memory) and the inability of the respondent to estimate the frequency, intensity, and duration of physical activity [62]. It was due to these considerations that we did not assess lifestyle physical activity, which is often sporadic, and even more difficult to recall and report than planned, discretionary, leisure-time exercise [63]. Here it should be noted that with regard to cost, staff training, participant burden, and time, self-reporting was the only feasible method for measuring PAL in such a large implementation programme [64]. It should also be noted that the cut-off point for physically active and inactive participants was not determined according to the CDC/ACSM physical activity recommendations [65], due to the limitations of the questionnaire. However, it has been suggested [7] that the amount of physical activity needed to prevent diabetes should in fact be set at lower than the recommendations for the general adult population given by CDC/ASCM.

## Conclusions

The present study shows that the vast majority of high risk individuals perceive the need to increase their PAL. However, apart from waist circumference, the perception of need appears not to be determined by the objective diabetes risk factors. Instead, the perception appears to be strongly determined by factors that are based on individuals’ subjective impressions. This finding, taken together with the association between physical activity awareness and a perceived need to increase PAL, highlights the importance of listening to and reflecting clients’ perceptions (which may not always be realistic), rather than focusing mainly on objective health measures. It is further important to increase clients’ awareness by *concretely* defining the association of physical activity with all the metabolic risk factors – and with the development of diabetes. This study did not assess the predictors of behaviour change, but it does demonstrate factors worth recognising in future interventions whose aim is to facilitate the intention and commitment to change physical activity behaviour among individuals at high risk of type 2 diabetes.

## Competing interests

The authors declare that they have no competing interests.

## Authors’ contributions

KV conceived of and designed the study, interpreted the data and drafted the manuscript. SS, JV, MV, PR and MEP participated in the design of the study. JV processed the data and performed the statistical analyses. All authors participated in interpretation of the results and critically revised subsequent versions of the paper for important intellectual content. TS, MV, MP, LN, LM, EK-H, SK-K, HO, and MEP were all in charge of the FIN-D2D concept and design, and the acquisition of the data. All the authors read and approved the final manuscript.

## Pre-publication history

The pre-publication history for this paper can be accessed here:

http://www.biomedcentral.com/1471-2458/12/514/prepub
